# Wait Times for Scheduling Appointments for Prevention of Macrovascular and Microvascular Complications of Diabetes: Cross-Sectional Descriptive Study

**DOI:** 10.2196/55351

**Published:** 2024-03-26

**Authors:** Corey H Basch, Grace C Hillyer, Charles E Basch

**Affiliations:** 1 Department of Public Health William Paterson University Wayne, NJ United States; 2 Department of Epidemiology, Mailman School of Public Health Columbia University New York, NY United States; 3 Department of Health Studies and Applied Educational Psychology Teachers College Columbia University New York, NY United States

**Keywords:** appointment scheduling, cardiologists, chronic disease, cross-sectional study, diabetes, endocrinologists, healthcare utilization, lifestyle modification, management plan, metabolic control, online booking, ophthalmologists, prevention, quality of life, scheduling appointments, scheduling, specialist, timeliness, United States, wait time, well-being

## Abstract

**Background:**

Diabetes is a chronic disease that requires lifelong management and care, affecting around 422 million people worldwide and roughly 37 million in the United States. Patients newly diagnosed with diabetes must work with health care providers to formulate a management plan, including lifestyle modifications and regular office visits, to improve metabolic control, prevent or delay complications, optimize quality of life, and promote well-being.

**Objective:**

Our aim is to investigate one component of system-wide access to timely health care for people with diabetes in New York City (NYC), namely the length of time for someone with newly diagnosed diabetes to obtain an appointment with 3 diabetes care specialists: a cardiologist, an endocrinologist, and an ophthalmologist, respectively.

**Methods:**

We contacted the offices of 3 different kinds of specialists: cardiologists, endocrinologists, and ophthalmologists, by telephone, for this descriptive cross-sectional study, to determine the number of days required to schedule an appointment for a new patient with diabetes. The sampling frame included all specialists affiliated with any private or public hospital in NYC. The number of days to obtain an appointment with each specialist was documented, along with “time on hold” when attempting to schedule an appointment and the presence of online booking capabilities.

**Results:**

Of the 1639 unique physicians affiliated with (private and public) hospitals in the 3 subspecialties, 1032 (cardiologists, endocrinologists, and ophthalmologists) were in active practice and did not require a referral. The mean wait time for scheduling an appointment was 36 (SD 36.4; IQR 12-51.5) days for cardiologists; 82 (SD 47; IQR 56-101) days for endocrinologists; and 50.4 (SD 56; IQR 10-72) days for ophthalmologists. The median wait time was 27 days for cardiologists, 72 days for endocrinologists, and 30 days for ophthalmologists. The mean time on hold while attempting to schedule an appointment with these specialists was 2.6 (SD 5.5) minutes for cardiologists, 5.4 (SD 4.3) minutes for endocrinologists, and 3.2 (SD 4.8) minutes for ophthalmologists, respectively. Over 46% (158/341) of cardiologists enabled patients to schedule an appointment on the web, and over 55% (128/228) of endocrinologists enabled patients to schedule an appointment on the web. In contrast, only approximately 25% (117/463) of ophthalmologists offered web-based appointment scheduling options.

**Conclusions:**

The results indicate considerable variation in wait times between and within the 3 specialties examined for a new patient in NYC. Given the paucity of research on wait times for newly diagnosed people with diabetes to obtain an appointment with different specialists, this study provides preliminary estimates that can serve as an initial reference. Additional research is needed to document the extent to which wait times are associated with complications and the demographic and socio-economic characteristics of people served by different providers.

## Introduction

Diabetes is a chronic disease that requires lifelong management and care, affecting around 422 million people worldwide [1] and roughly 37 million in the United States (1 in 10 Americans) [2]. The 3 main kinds of diabetes are type 1, an autoimmune disease in which the body stops producing insulin; type 2, where the body stops using insulin well and can no longer regulate normal glucose levels; and gestational diabetes, which occurs during pregnancy. In the United States, approximately 90%-95% of people affected have type 2 diabetes [2], which is the most expensive chronic condition [3], costing an estimated US $327 billion in 2017.

Patients newly diagnosed with diabetes must work with health care providers to formulate a management plan, including lifestyle modifications and regular office visits, to improve metabolic control and associated long-term disease outcomes, prevent or delay complications, optimize quality of life, and promote well-being. In addition to attaining stable metabolic control through lifestyle changes, diabetes self-care clinical management also includes weight management and cardiovascular and retinopathy risk monitoring [4]. According to the Standards of Care in Diabetes 2023, treatment decisions must be timely, include social support, and be formulated collaboratively between patients and their health care providers, with consideration of the individual’s preferences, prognoses, comorbidities, and financial factors [5]. Defined as “the system’s capacity to provide care quickly after a need is recognized [6], timeliness in health care is associated with insurance type [7,8] and a lack thereof has been shown to delay treatment initiation [7,9,10].

Therapeutic inertia, or “lack of timely adjustment to therapy when a patient’s treatment goals are not met” [11], can be dire, yet research demonstrates it remains prevalent in Western countries [12]. Delays in treatment are known to cause more severe complications [13-16], including hypertension and cardiovascular disease, nonperipheral and peripheral retinopathy, nephropathy, and peripheral vascular diseases [10,17]. Unintentional delays in diagnosis further complicate screening and treatment and are medically damaging [18].

Timely care begins with initial appointments at the time of diagnosis. There are gaps between high-quality health care and what patients actually receive, and more timely care is one of several key focal points for improvement [4]. Overcoming population-wide delays in assessment, monitoring, and treatment is complex and involves interactions among patients, health care providers, and the health care system [19,20]. Effective interventions to decrease hyperglycemia and improve glycemic control remain a major challenge [21-23]. Therefore, researchers suggest that monitoring systems that provide a holistic view of the quality of care are necessary to combat this issue [20].

The purpose of this study was to investigate one component of system-wide access to timely health care for people with diabetes in New York City (NYC), namely the length of time for someone with newly diagnosed diabetes to obtain an appointment with 3 diabetes care specialists: a cardiologist, an endocrinologist, and an ophthalmologist, respectively. As type 1 and type 2 diabetes require similar specialist management, we do not differentiate between the types but refer to diabetes as a single entity for the purposes of this study. The sampling frame included all specialists affiliated with any private or public hospital in NYC in 2022. We documented the number of days to obtain an appointment with each specialist. Secondary objectives were to describe “time on hold” when attempting to schedule an appointment and the presence of web-based booking capabilities.

## Methods

### Design

The design of this study was cross-sectional, and the study was limited to a descriptive analysis.

### Study Setting

The offices of three different kinds of specialists—cardiologists, endocrinologists, and ophthalmologists—in NYC were contacted by telephone to determine the number of days required to schedule an appointment for a new patient with diabetes.

### Sampling, Eligibility Criteria, and Data Collection

The sample frame was cardiologists, endocrinologists, and ophthalmologists, listed on the websites of all accredited private or public hospitals in NYC in the fall of 2022.

Each website was reviewed, and contact information for each provider was excerpted into a separate file. Each office telephone number listed on the website was called to determine the following information: whether the physician was accepting new patients, type of insurance accepted (private, public, and none), and number of days for a person with newly diagnosed diabetes to obtain an appointment.

Exclusion criteria included physicians who were not accepting new patients, no longer worked at the hospital, those who only saw specific types of patients (eg, pediatrics), and those who did not have a working phone number. Furthermore, when a doctor was listed at multiple locations, a random number generator was used to randomly select only one office to include. Time on hold while scheduling appointments and availability of web-based booking (“ZocDoc” or the hospital website) were noted, as well as the type of insurance accepted by providers (Medicaid, commercial, or none).

### Statistical Analysis

**Figure 1:**
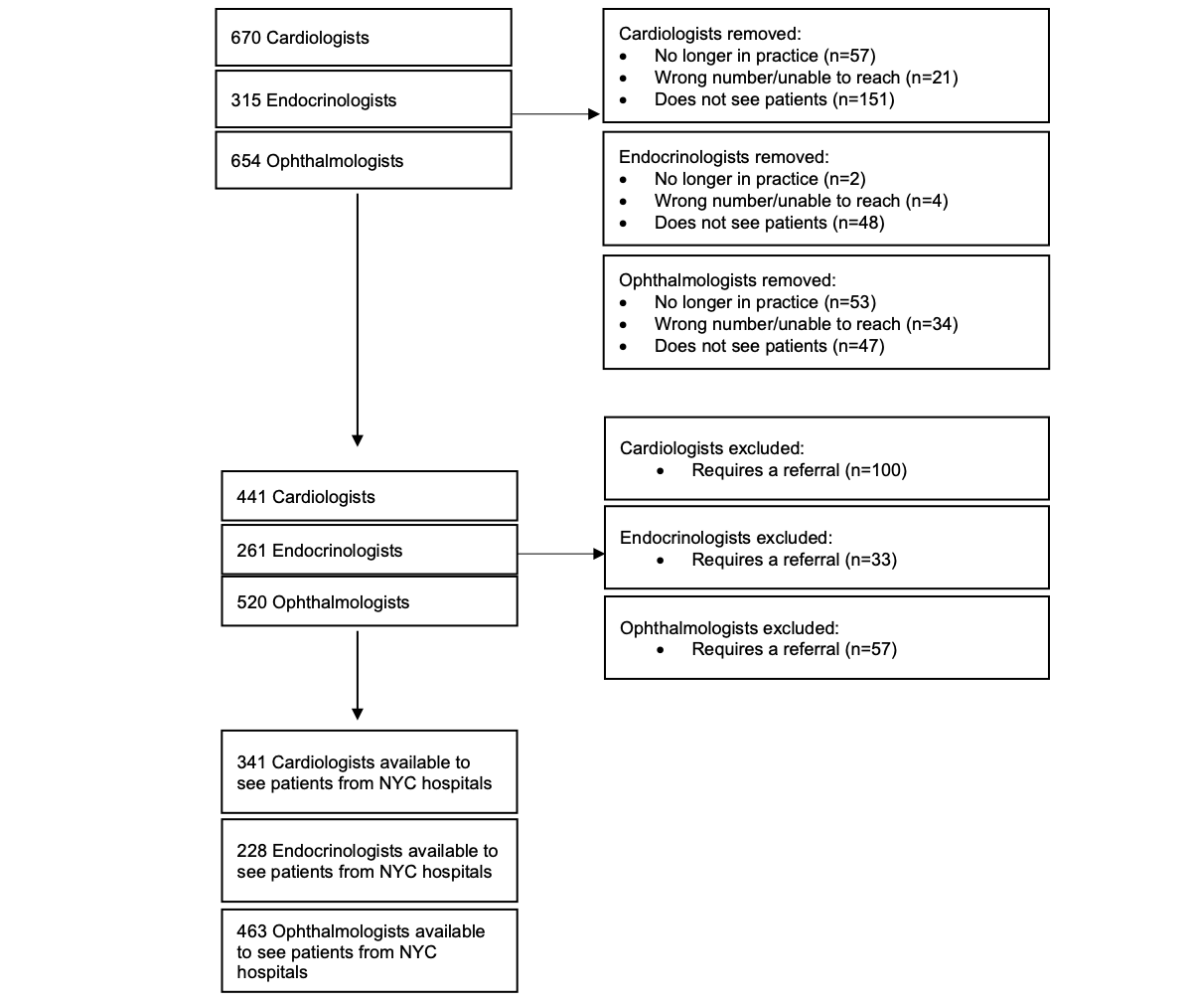
Diabetes specialists affiliated with New York City (NYC) hospitals.

Of the 1639 unique physicians affiliated with (private and public) hospitals in the three subspecialties, a total of 1032 (341 of 670 cardiologists, 228 of 315 endocrinologists, and 463 of 654 ophthalmologists) were in active practice and did not require a referral (Figure 1). For each subspecialty, we determined the mean (SD), IQR, and total range for the continuous variables: the number of days until the first available appointment and the time to reach someone at the office on the telephone to make an appointment. Categorical variables included types of insurance accepted and whether it was possible to schedule an appointment through ZocDoc (a NYC-based web-based appointment booking service) or on the web at the hospital website. All analyses were performed using SPSS (version 28; IBM Corp) [21].

### Ethical Considerations

This study was approved by the institutional review board at Teachers College, Columbia University (protocol #23-029). This study did not meet the definition of human participant research for the institutional review board at William Paterson University.

## Results

The mean wait time for scheduling an appointment was 36 (SD 36 days; IQR 12.0-51.5) days for cardiologists; 82 (SD 47; IQR 56.0-101.0) days for endocrinologists; and 50.4 (SD 56; IQR 10.0-72.0) days for ophthalmologists (Table 1). The median wait time was 27.0 days for cardiologists, 72.0 days for endocrinologists, and 30.0 days for ophthalmologists. The mean time on hold while attempting to schedule an appointment with these specialists was 2.6 (SD 5.5) minutes for cardiologists, 5.4 (SD 4.3) minutes for endocrinologists, and 3.2 (SD 4.8) minutes for ophthalmologists, respectively (Table 1). Over 46% (158/341) of cardiologists enabled patients to schedule an appointment on the web (38/341, 11.1% on ZocDoc and 120/341, 35.2% on their website), and over 56% (128/228) of endocrinologists enabled patients to schedule an appointment on the web (52/228, 22.8% on ZocDoc and 76/228, 33.3% on their website). In contrast, only approximately 25% (117/463) of ophthalmologists offered web-based appointment scheduling options (43/463, 9.3% on ZocDoc and 74/463, 16.0% on their website).

**Table 1:** Characteristics of timeliness of care among New York City specialists (N=1032).

			Cardiologists (n=341)		Endocrinologists (n=228)		Ophthalmologists (n=463)
**Time on hold (minutes)**							
	Mean (SD)	2.6 (5.5)		5.4 (4.3)		3.2 (4.8)	
	Range	<1-48		<1-24.2		<1-66	
**Time until appointment (days)**							
	Mean (SD)	36 (36)		82 (47)		50.4 (56)	
	Median (IQR)	27.0 (12.0-51.5)		72.0 (56.0-101.0)		30.0 (10.0-72.0)	
**Insurance accepted, n (%)**							
	Private only	9 (2.6)		14 (6.1)		5 (1.1)	
	Private and Medicare	44 (12.9)		79 (34.6)		108 (23.3)	
	Private, Medicare, and Medicaid	285 (83.6)		131 (57.5)		349 (75.4)	
	None	3 (0.9)		4 (1.8)		1 (0.2)	
Appointments available on ZocDoc, n (%)			38 (11.1)		52 (22.8)		43 (9.3)
Appointments available on website, n (%)			120 (35.2)		76 (33.3)		74 (16.0)

## Discussion

### Principal Results

The importance of early detection and treatment of macrovascular and microvascular complications caused by diabetes results in considerable personal and societal costs [22,23]. Living with diabetes requires considerable self-management on a daily basis, as well as scheduling multiple clinical appointments for screening and checkups. Because of the increased risks of macrovascular and microvascular complications, diabetes is the most costly chronic condition in the United States [24], with some estimates stating that 25% of all US health care costs are attributed to the disease [25]. Timely access to health care and education at an early stage in the natural history of the disease can help people with diabetes learn self-management knowledge and skills and delay the incidence of complications [5,26]. Surprisingly, we did not identify any published studies in peer-reviewed biomedical journals that documented the time required for a new patient to obtain an appointment with each of the 3 main clinical specialists who care for people with diabetes: cardiologists, endocrinologists, and ophthalmologists.

The results from this study provide an initial estimate of the wait times required for a new patient to see each of the 3 specialists in NYC. The results indicate considerable variation in wait times both between and within the 3 specialties examined. For example, compared with the mean wait time for a new patient to see a cardiologist, the mean wait time to see an endocrinologist was more than twice as long (36 days vs 82 days), with the mean wait time for seeing an ophthalmologist falling in between (approximately 50 days). The 25% of cardiologists with the shortest wait times were 12 or fewer days, while the 25% with the longest wait times were 51.5 days or longer. For endocrinologists, the 25% with the shortest wait times were 56 or fewer days, while the 25% with the longest wait times were 101 days or longer. The largest IQR in wait times was observed for ophthalmologists; the 25% with the shortest wait times was 10 or fewer days, while the 25% with the longest wait times was 72 days or longer.

While the concept of surveillance may have a negative connotation from socio-political perspectives, surveillance is one of the main public health strategies used for disease prevention and control. By identifying subgroups within the population at high risk, scarce resources can be directed in an appropriate and efficient way. Disease surveillance, therefore, plays a prominent role in national disease prevention and health promotion efforts, including documentation of causes of death, comorbidity, disability, the use and cost of health care, and a wide range of preventive health behaviors [27,28]. Nevertheless, we did not identify any published studies in peer-reviewed biomedical literature regarding wait times for medical appointments, a gap in current knowledge this study aimed to address.

### Limitations

As this was a cross-sectional study, the findings cannot be generalized over time. There were some inaccuracies on the web pages used to construct the sampling frame (including physicians listed who did not provide clinical care or were no longer employed at the respective clinical setting). This in itself is an important finding because it shows the frustration a person with diabetes may face in attempting to schedule an appointment with a provider who is not available. Another limitation is that the data were based on reports from office staff, which may not have been accurate. Nevertheless, this simulates the kinds of information that would have been available to prospective patients calling the respective offices. The doctors included in this study were all affiliated with a hospital in NYC, which provides an incomplete sampling frame of all practices.

### Conclusions

This study was focused on health care providers addressing cardiology (macrovascular complications), endocrinology (metabolic control), and ophthalmology (microvascular complications causing diabetic retinopathy) [29]. These 3 aspects of care influence the natural history of diabetes and its major complications. Endocrine-level control of metabolism is paramount for those with diabetes, as it mediates the progression of disease [29]. Poor metabolic control over time causes a wide range of negative clinical consequences [29], including increased risk for heart disease and heart failure [30], which is the leading cause of death in the United States, as well as increased risk, progression, and severity of diabetic retinopathy, the leading cause of nontraumatic blindness among working age adults [30,31]. Both cardiovascular disease and diabetic retinopathy can be treated effectively in the early stages; however, once clinical signs and symptoms are apparent, treatment is less effective [29,30].

Given the paucity of research on wait times for newly diagnosed people with diabetes to obtain an appointment with different specialists, this study provides preliminary estimates that can serve as an initial reference. The geographic scope of this study was limited to NYC, which has a far greater concentration of clinical specialists than many other geographic areas [32]. It is reasonable to expect that wait times may be considerably longer in areas with fewer providers. However, this requires confirmation since providers in some areas may offer remote accessibility. Those with the greatest needs and at the highest risk of experiencing complications from diabetes tend to have the least access to timely health care services [33-35]. While time spent waiting for an appointment is relevant for everyone in the population, it is especially important for people who require multiple annual appointments [36,37]. Additional research is needed to document the extent to which wait times are associated with complications and the demographic and socio-economic characteristics of people served by different providers.

## Abbreviations

NYC: New York City

## Data Availability

The data sets generated during and analyzed during this study are available upon reasonable request.
